# Older Adult Refugees’ Roles in Creating Partnerships for Culturally Responsive Healthcare

**DOI:** 10.1093/geroni/igaa057.1208

**Published:** 2020-12-16

**Authors:** Denise Lewis, Desiree Seponski, Amber Kelley

**Affiliations:** 1 University of Georgia, Athens, Georgia, United States; 2 University of Georgia, University of Georgia, United States

## Abstract

The aim of this presentation is to reveal the importance of engaging older refugee community members in creating partnerships with local healthcare providers to implement culturally responsive interventions. Such engagement invites older refugees’ participation and encourages continued connection to country of origin beliefs and culture, particularly within the sphere of healthcare and medical family therapy. Cambodian and Laotian refugee families in coastal Alabama were interviewed via qualitative community-based participatory research and ethnographic, in-depth interviews focused on familial and communal processes. Local healthcare providers engaged in focus group discussions regarding cultural processes associated with health beliefs and behaviors and in periodic outreach through culturally responsive health fairs. We found that older adults play important roles in refugee populations as community leaders, problem-solvers, and legacy-carriers upholding traditional values and cultural continuity. They also maintain and promote continuity by employing traditional, complementary, or alternative medicine (TCAM). Recursive processes also emerged as older individuals sought care from younger community members in times of sickness, including having younger generations provide language translation and transportation to local healthcare centers and hospitals and in navigating the United States healthcare system. In addition, community members joined with local healthcare providers to aide in health and healthcare literacy among refugees and to educate local physicians on culturally responsive interventions. Implications include the inclusion of older adults in health decisions and the promotion and maintenance of community partnerships with health agencies that both encourage TCAM utilization and also allow for access to ongoing, appropriate treatment within the US healthcare system.

